# Supplementary material for: The introduction of genetic counseling in Ethiopia: Results of a training workshop and lessons learned
Source: PLoS One. 2021 Jul 23;16(7):e0255278. doi: 10.1371/journal.pone.0255278 (PMC8301664; doi:10.1371/journal.pone.0255278)
Supplement: S1 Assessment — (DOCX) [file pone.0255278.s001.docx]

MiGene Training Program Post-assessment

1. Which of the following is TRUE regarding genes and human disease?
   1. All changes to a gene’s sequence cause a genetic disease
   2. Only mutations which involve multiple genes can cause a genetic disease
   3. Some, but not all, changes to a gene cause a genetic disease
   4. Genetic diseases are always inherited from a parent
2. When first delivering the bad news to a family, you should be very organized and plan to make it as quick a process as you can.
   1. True
   2. False

Please explain:

1. A karyotype is most useful at diagnosing which of the following conditions?
   1. Trisomy 21/Down syndrome
   2. Sickle cell anemia
   3. Neural tube defects
   4. Congenital heart defects
2. Empathy is an innate reaction and cannot be learned. You either have it or you don’t.
   1. True
   2. False

Please explain:

1. Which of the following is TRUE regarding chromosomes?
   1. Humans have 26 pairs of chromosomes for a total of 52 chromosomes per cell
   2. Chromosomes are made up of tightly coiled DNA
   3. Males have two X-chromosomes
   4. Females have one X-chromosome and one Y-chromosome
2. An empathetic response from a genetic counselor helps a patient manage their emotions.
   1. True
   2. False

Please explain:

1. When a new genetic diagnosis is made in a family, it is common for the parents to want to keep this a secret.
   1. True
   2. False

Please explain:

1. Which of the following best describes the medical problems experienced by patients with Trisomy 21/Down syndrome?
   1. Normal intellect, congenital heart malformations, and normal growth
   2. Intellectual disability, congenital heart malformations and growth deficiencies
   3. Neural tube defects, brain malformations, and normal growth
   4. Abnormal facial features, normal intellect, and congenital heart malformations
2. When delivering bad news to a patient/family you should try to help them identify other people who they can talk to after the appointment.
   1. True
   2. False

Please explain:

1. Which of the following types of genetic disease are more likely in children from consanguineous parents?
   1. Autosomal dominant
   2. Autosomal recessive
   3. X-linked dominant
   4. X-linked recessive
2. In a family with a new genetic diagnosis, it is common for parents and grandparents to feel that they are to blame.
   1. True
   2. False

Please explain:

1. Which of the following is TRUE regarding neural tube defects?
   1. All forms of neural tube defects cause death prior to delivery
   2. Neural tube defects can be caused by maternal folic acid deficiency
   3. There is no increased risk of neural tube defects for future pregnancies of parents of a child with a neural tube defect
   4. All patients with neural tube defects will have abnormalities found with genetic testing
2. An empathic response from a genetic counselor can help a client/patient make sense of the information the genetic counselor has provided.
   1. True
   2. False

Please explain:

1. Which of the following best describes how DNA, proteins, and genes functions in the human body?
   1. Genes are made up of DNA and are used to make proteins
   2. Genes are made up of protein and are used to make DNA
   3. Proteins are made up of genes
   4. DNA is responsible for most of the work in cells and is required for the structure, function, and regulation of the body’s tissues and organs
2. The best way to show empathy is for the genetic counselor to display an emotional reaction to genetic/medical news that is the same as the patient’s.
   1. True
   2. False

Please explain:

1. Which of the following is TRUE regarding family history/pedigree collection?
   1. Should be collected on all patients seen for a genetic counseling visit
   2. Assists providers in determining the inheritance pattern present in a patient/family with a genetic disease
   3. Should contain 3-generations of the family
   4. All of the above
2. Which of the following conditions is a multifactorial disorder?
   1. Trisomy 21/Down syndrome
   2. Cystic fibrosis
   3. Sickle cell anemia
   4. Neural tube defect
3. Both genetic counselors and geneticists perform physical exams on their patients.
   1. True
   2. False

Please explain:

1. Which of the following is TRUE regarding genetic testing?
   1. Can be used to confirm a suspected genetic disease
   2. Can be used to rule out a genetic disease
   3. Prenatal genetic testing is used to determine if a fetus is affected with a genetic disease
   4. All of the above
2. When a patient displays an extreme emotional reaction like anger to genetic information, it is often because the patient is trying to cope with information that they did not want to hear.
   1. True
   2. False

Please explain:

1. Empathy is the process of communicating an understanding of what a patient is experiencing.
   1. True
   2. False

Please explain:

1. For an individual with an autosomal dominant genetic disease, what is their risk of passing on the condition to their next child?
   1. Very low
   2. 25%
   3. 50%
   4. 100%
2. Genetic counselors help patients and their families understand why a disease has occurred in their child and/or family
   1. True
   2. False

Please explain:

1. Empathy and sympathy have the same impact on a patient’s emotional state.
   1. True
   2. False

Please explain:

1. Which of the following conditions is a single-gene disorder?
   1. Trisomy 21/Down syndrome
   2. Sickle cell anemia
   3. Trisomy 13/Patau syndrome
   4. Neural tube defects
2. Which of the following is TRUE regarding Trisomy 21/Down syndrome?
   1. Most parents of a child with Trisomy 21/Down syndrome have a significantly elevated risk (>50% chance) of having another child with Trisomy 21/Down syndrome with future pregnancies
   2. Trisomy 21/Down syndrome follows an autosomal recessive inheritance pattern
   3. Only those families with a translocation-type Trisomy 21/Down syndrome have an increased risk for Trisomy 21/Down syndrome with future pregnancies
   4. Only mothers over 35 years of age are at risk of having a child with Trisomy 21/Down syndrome
3. The best way to be empathetic is by telling the patient that you have put yourself in their shoes and can really understand what they are experiencing.
   1. True
   2. False

Please explain:

1. When a patient displays an extreme emotional reaction like anger to genetic information, it is often because the genetic counselor did a poor job explaining the result.
   1. True
   2. False

Please explain:

1. When delivering bad news to a patient/family you should try to anticipate how they will respond.
   1. True
   2. False

Please explain:

1. Which of the following is TRUE regarding congenital heart defects?
   1. Most congenital heart defects are caused by multiple genetic and environmental reasons
   2. Congenital heart defects follow an autosomal dominant inheritance pattern
   3. Congenital heart defects follow an autosomal recessive inheritance pattern
   4. All patients with congenital heart defects have a parent also affected with a congenital heart defect
