# Supplementary material for: The introduction of genetic counseling in Ethiopia: Results of a training workshop and lessons learned
Source: PLoS One. 2021 Jul 23;16(7):e0255278. doi: 10.1371/journal.pone.0255278 (PMC8301664; doi:10.1371/journal.pone.0255278)
Supplement: S1 Checklist — (DOCX) [file pone.0255278.s002.docx]

|  | **Completed?** | **Comments** |
| --- | --- | --- |
| **Identified location where counseling will be held** | **Y N N/A** |  |
| **Confirmed the appropriate family members were present for counseling** | **Y N N/A** |  |
| **Identified and discussed the patient’s/family’s goals for session** | **Y N N/A** |  |
| **Provided education for indication** | **Y N N/A** |  |
| **Provided empathic counseling (1-3 psychosocial statements)** | **Y N N/A** |  |
| **Obtained a 3-generation pedigree** | **Y N N/A** |  |
| **Evaluated family history and convened additional risk information as appropriate** | **Y N N/A** |  |
| **Discussed all treatment options** | **Y N N/A** |  |
| **Discussed next steps with patient and family** | **Y N N/A** |  |
| **Provided family with appropriate resources** | **Y N N/A** |  |
| **Acted in accordance with all legal and ethical guidelines** | **Y N N/A** |  |
