# Supplementary material for: The introduction of genetic counseling in Ethiopia: Results of a training workshop and lessons learned
Source: PLoS One. 2021 Jul 23;16(7):e0255278. doi: 10.1371/journal.pone.0255278 (PMC8301664; doi:10.1371/journal.pone.0255278)
Supplement: Attachment — Submitted filename: Response to Reviewers.docx [file pone.0255278.s003.docx]

C.S. Mott Children’s Hospital

Michigan Medicine

**Department of Pediatrics**

**Division of Pediatric Genetics,**

**Metabolism & Genomic Medicine**

Jeffrey W. Innis, M.D., Ph.D., Interim Director	Adelyn Beil, M.S., MPH., CGC

Ayesha Ahmad, M.D. Lauren DeMeyer, M.S.

Elizabeth Ames, M.D., Ph.D. Rachel Fisher, M.S., CGC

Thomas Glover, Ph.D.	Megan Glassford, M.S., CGC

Mark C. Hannibal, M.D., Ph.D.	Susan L. Lipinski, MPH, RD

Catherine E. Keegan, M.D., Ph.D.	Bridget C. O’Connor, M.S., CGC

Kristen N. Lee, M.D.	Jessica Omark, M.S., CGC

Donna M. Martin, M.D., Ph.D.	Beverly M. Yashar, M.S., CGC, Ph.D.

Amanda B. Pritchard, M.D.	Shane C. Quinonez, M.D. Marwan K. Tayeh, Ph.D.	Jess. G. Thoene, M.D. Chen Yang, M.D., Ph.D.

June 25, 2021

Dear Dr. Suppiah,

We are pleased to be resubmitting our manuscript titled, “The Introduction of Genetic Counseling in Ethiopia: Results of a Training Workshop and Lessons Learned” following the requested revisions. We feel the additional comments have further improved the manuscript. All comments and suggestions have been addressed in our resubmission. Our response to each point is listed below and how it was addressed including the specific text added. We look forward to your review of our revision. Thank you again for your time.

Sincerely,

Shane C. Quinonez, MD

Bridget C O’Connor MS, CGC

Michelle F Jacobs MS, CGC

Atnafu Mekonnen MD

Ayalew Marye MD

Delayehu Bekele MD, MPH

Beverly M Yashar MS, PhD

Erika Hanson BA

Abate Yeshidinber MD

Getahun Wedaje

**Response to Reviewers**

Reviewer #1 Comments:

***Reviewer***: Keep abbreviations throughout the text.

***Response****:* Thank you for point this out. This has been addressed throughout the manuscript.

***Reviewer****:* It should interesting to know the interval of time the surveys (pre and post workshops) were completed in both cohorts

***Response****:* We have added this information to the Materials and Methods section. We have specifically added the following text:

“For Cohort 1 and Cohort 2 pre-assessments were completed on the first day of the workshop. Individual lecture evaluations were completed at the end of each day with the entire curriculum evaluation and post-assessment completed at the end of the final day of the workshop”

***Reviewer****:* Although the statistical analyzes are ok, I think they could be more complete, for example, what cases Welch's correction was necessary.

***Response****:* We have revised this section of the Materials and Methods section. Upon further review of the analyses used in the paper we found Welch’s correction was not used for any of the data presented so this was removed. The section now reads as follows:

“Assessment data and survey responses were collected in Excel. R was used to calculate pooled Student’s t-tests to assess statistically significant differences between pre- and post-assessments for each competency

***Reviewer****:* Figure 1 is difficult to read and should be attached again.

***Response****:* This has been reuploaded as a higher resolution document. If still blurry the downloadable version should be clearer. Thank you for pointing this out.

Reviewer #2 Comments:

***Reviewer:*** In the first paragraph of page 3, when describing the medical genetics need in Ethiopia, there is no information about the current services that are available for example, number of clinical genetics services, number of clinical geneticist and genetics counsellors (if any) and the population size. This would be useful to explain current practice in the country.

***Response:*** This information has been added. We have added the following text:

“Currently there are no established medical genetics services with no in-country clinical geneticists or genetic counselors.

***Reviewer:*** Line 138 : .. .."Ethiopian physician who translated confusing topics to trainee ": please mention what language was used.

***Response:*** This information was added. Specifically, the following text is now in the Materials and Methods section:

“Since the Ethiopian medical curriculum is taught in English, the curriculum was delivered in English by three study authors (SQ, BO, MJ) who were aided by an Ethiopian physician who translated confusing topics to trainees and/or questions to the trainers in Amharic, the principal language in Ethiopia. While medical education in Ethiopia is generally taught in English, Amharic and Afan Oromo are the most commonly spoken languages at SPHMMC with patients.”

***Reviewer***: Line 140 : " Attempts were made to choose nurses with adequate English fluency" - to understand possible language barriers, please describe the language(s) that is commonly used by the nurses in clinic. This was also mentioned as a barrier in line 274.

***Response***: In addition to the text shown above additional information was added. The full section includes the following text:

“Since the Ethiopian medical curriculum is taught in English, the curriculum was delivered in English by three study authors (SQ, BO, MJ) who were aided by an Ethiopian physician who translated confusing topics to trainees and/or questions to the trainers in Amharic, the principal language in Ethiopia. While medical education in Ethiopia is generally taught in English, Amharic and Afan Oromo are the most commonly spoken languages at SPHMMC with patients. Attempts were made to choose nurses with adequate English fluency for ease of training.”

**Reviewer**: Line 146 / Line 147 and line 357 to 359 :".. decrease the content on covering Western-based genetic counselling principles." It was mentioned that these principles were the most difficult to grasp by nurses and were more likely to misalign with established Ethiopian cultural norms. Which principles are difficult and what is the established cultural norms that is is misaligned with? It is not clear as the Training Programme Assessment covered broadly on empathy, confidentiality and delivery of bad news. This was also mentioned as a barriers in line 274. Please explore this part about Western genetic counselling principles that are misaligned.

**Response**: This is an important point. To address this, we included the following text which included additional references now in the manuscript:

“The three topics removed for Cohort 2 were Facilitated Decision-making, Delivering Bad News and Handling Patient Emotion. Though these areas of practice are used regularly in Ethiopia, previous work has shown differences between Western genetic counseling practice and practices in Ethiopia surrounding patient-provider communication, patient autonomy and decision-making especially [24,25]. For example, previous work in Ethiopia showed 40% of all medical decisions in rural Ethiopian patients were made by a woman’s husband [26]. “

**Reviewer:** Under Results - Line 200 : "the genetic counselling content specific scores" : the questions in the assessment for genetic counselling content such as Q2 / Q4 / Q6 and others are True/False but followed by 'please explain'. Are there any scores given to the explanation or it is for collection of field notes?

**Response:** This information was added to the Materials and Methods section. The following

text was added:

“For True/False assessment questions though individuals were able to provide an

explanation for their True/False response this aspect of the question was not scored.”

**Reviewer:** The practical barriers were well described in the discussion section and some good suggestions were given moving forward. Line 377 : "This works shows the feasibility of training nurses in the medical knowledge necessary to discuss various medical genetics topics with patients and families.". The result has shown an improvement in medical genetics knowledge and good feedback from the nurses trained but the logistical and other barriers have made it not feasible to train nurses using this 'Train the Trainer' method? Please comment.

**Response:** This important point was addressed with the following text added to the Discussion:

“It is also important to note our initial intent was for our workshops to follow a Train the Trainer model. While we did incorporate two trainees from Cohort 1 as trainers for Cohort 2 we are uncertain of the ability or comfort of Cohort 2 participants to train others. The reasons for this are multifactorial and include many of the barriers outlined in Table 3 as well as the new and complex subject material and uncertainties regarding the minimum level of competency needed to function as an effective trainer. Additional work will be required to enable trained nurses to competently and comfortably function as future trainers.”
